# Increased Needle Nitrogen Contents Did Not Improve Shoot Photosynthetic Performance of Mature Nitrogen-Poor Scots Pine Trees

**DOI:** 10.3389/fpls.2016.01051

**Published:** 2016-07-20

**Authors:** Lasse Tarvainen, Martina Lutz, Mats Räntfors, Torgny Näsholm, Göran Wallin

**Affiliations:** ^1^Department of Forest Ecology and Management, Swedish University of Agricultural SciencesUmeå, Sweden; ^2^Department of Biological and Environmental Sciences, University of GothenburgGothenburg, Sweden

**Keywords:** arginine, *J*_max_, optimality, phosphorus, photosynthesis, *Pinus sylvestris*, resource-use efficiency, *V*_cmax_

## Abstract

Numerous studies have shown that temperate and boreal forests are limited by nitrogen (N) availability. However, few studies have provided a detailed account of how carbon (C) acquisition of such forests reacts to increasing N supply. We combined measurements of needle-scale biochemical photosynthetic capacities and continuous observations of shoot-scale photosynthetic performance from several canopy positions with simple mechanistic modeling to evaluate the photosynthetic responses of mature N-poor boreal *Pinus sylvestris* to N fertilization. The measurements were carried out in August 2013 on 90-year-old pine trees growing at Rosinedalsheden research site in northern Sweden. In spite of a nearly doubling of needle N content in response to the fertilization, no effect on the long-term shoot-scale C uptake was recorded. This lack of N-effect was due to strong light limitation of photosynthesis in all investigated canopy positions. The effect of greater N availability on needle photosynthetic capacities was also constrained by development of foliar phosphorus (P) deficiency following N addition. Thus, P deficiency and accumulation of N in arginine appeared to contribute toward lower shoot-scale nitrogen-use efficiency in the fertilized trees, thereby additionally constraining tree-scale responses to increasing N availability. On the whole our study suggests that the C uptake response of the studied N-poor boreal *P. sylvestris* stand to enhanced N availability is constrained by the efficiency with which the additional N is utilized. This efficiency, in turn, depends on the ability of the trees to use the greater N availability for additional light capture. For stands that have not reached canopy closure, increase in leaf area following N fertilization would be the most effective way for improving light capture and C uptake while for mature stands an increased leaf area may have a rather limited effect on light capture owing to increased self-shading. This raises the question if N limitation in boreal forests acts primarily by constraining growth of young stands while the commonly recorded increase in stem growth of mature stands following N addition is primarily the result of altered allocation and only to a limited extent the result of increased stand C-capture.

## Introduction

Many studies, since the mid-1900's, have shown how addition of nitrogen (N) to boreal, coniferous forests results in sharply increased basal area increment rates (Tamm, 1956; Brix, 1971, 1983; Linder and Axelsson, 1982; Axelsson and Axelsson, 1986; Linder, 1987). This well-known effect of N addition is also evidence for the general notion that such forests are N-limited (e.g., Tamm, 1991). Two interacting processes can explain the higher aboveground growth rates following N addition; increased canopy-scale photosynthesis and shifts in partitioning between the above- and belowground carbon (C) pools. Canopy-scale photosynthetic enhancements can be achieved either by higher needle N contents, owing to the central importance of N for Calvin cycle and thylakoid proteins (Evans, 1989), or by production of additional leaf area or both. Thus, the balance between photosynthetic capacity and leaf area responses may have important implications on how N addition affects the needle- and canopy-scale light- and nitrogen-use efficiencies. Moreover, the relative contributions to the observed aboveground growth response by enhanced canopy-scale C capture and by C partitioning shifts will likely depend on both needle-scale photosynthetic performance responses and stand age. While N responses of boreal conifers have been extensively studied with respect to stand growth (e.g., Tamm, 1956; Axelsson and Axelsson, 1986; Linder, 1987; Lim et al., 2015) and short-term needle photosynthetic capacities (e.g., Kellomäki and Wang, 1997; Laitinen et al., 2000; Sigurdsson et al., 2002), surprisingly few studies have investigated the long-term canopy-scale, and within-canopy, photosynthetic performance responses to N fertilization based on observations made under field conditions (but see Brix, 1971; Linder and Axelsson, 1982). Rather, canopy-scale estimates of photosynthetic performance in temperate and boreal stands are commonly based on a relationship between leaf-scale photosynthetic capacity and leaf N content. The use of N as a scaling factor is supported by observational data showing photosynthetic capacity variation in response to foliar N contents (e.g., Evans, 1989; Reich et al., 1995; Kellomäki and Wang, 1997; Niinemets et al., 2001; Han et al., 2004; Wyka et al., 2012), and by theoretical calculations indicating that canopy-scale photosynthesis is maximized when N is allocated proportionally to photosynthetic photon flux density (*Q*) within the canopy (Field, 1983; Farquhar, 1989). However, this proportionality is not observed in natural canopies and various explanations have been offered for why the theoretically optimal pattern does not emerge. These include within-leaf N allocation patterns (Hikosaka and Terashima, 1995, 1996), limitations due to leaf morphology (Dewar et al., 2012), constraints on hydraulic capacity (Peltoniemi et al., 2012; Buckley et al., 2013), and the importance of model assumptions regarding the within-canopy variation in light availability and quality (Hikosaka, 2014, 2016). While modeling studies have shown optimal N allocation to have the ability to considerably increase the plant-scale C uptake in some cases (Hirose and Werger, 1987; Hollinger, 1996), the predicted differences in C uptake between observed and optimal N allocation patterns were small for hemi-boreal *Picea abies* growing under N-rich conditions (Tarvainen et al., 2013).

High N deposition rates may lead to unbalanced nutrient availabilities and the observed potential of phosphorus (P) to constrain leaf-scale photosynthetic capacity (Reich and Schoettle, 1988; Niinemets et al., 2001; Bauer et al., 2004; Bown et al., 2007; Reich et al., 2009; Walker et al., 2014; Ellsworth et al., 2015) suggests that some of the observed non-optimality in N allocation under N-rich conditions could be caused by insufficient foliar P contents. This possibility is supported by a recent Europe-wide study reporting deteriorating foliar P concentrations in response to N deposition for several species, including *Pinus sylvestris* (Jonard et al., 2015), and by reports of P limitation of ecosystem productivity (Peñuelas et al., 2013) and forest growth (Gradowski and Thomas, 2006; Prietzel et al., 2008; Braun et al., 2010) in regions commonly considered N-limited. Foliar P content has been included in some recent models of leaf-scale photosynthetic capacity (Domingues et al., 2010) and forest productivity (Mercado et al., 2011) developed for the tropical region where P limitation is thought to be common (Vitousek, 1984). However, foliar P contents are rarely reported from photosynthetic studies in boreal ecosystems (but see Reich et al., 2009; Tarvainen et al., 2013), perhaps due to the predominance of N-poor soils, and data of potential P effects on C assimilation are lacking from this region (Walker et al., 2014).

Leaf photosynthetic capacity could also become decoupled from foliar N content in response to increasing N allocation to non-photosynthetic compounds such as free amino acids with increasing N availability (Näsholm and Ericsson, 1990; Näsholm, 1994). Arginine synthesis has been suggested to detoxify leaf tissues of ammonia (Rabe and Lovatt, 1984, 1986) and, accordingly, arginine commonly dominates the needle free amino acid N pool in conifers growing under N-rich conditions (Näsholm and Ericsson, 1990; Nordin et al., 2001; Bauer et al., 2004). While arginine synthesis often occurs in response to low P availability, it may be caused by deficiencies in several macro- and micronutrients (Rabe and Lovatt, 1984) and has been argued to be a general response to growth reducing stresses rather than a response to the availability of any specific nutrient (Näsholm and Ericsson, 1990). Excess N may also be stored in photosynthetic compounds as suggested by Warren et al. (2003) who found in their study of temperate *P. sylvestris* that <7% of the total N was in free amino acids or amides, and suggested that Rubisco functioned as a storage protein for N in addition to its role in C assimilation. The importance of arginine as a long-term N store in needle tissues is reduced because arginine-bound N is not readily retranslocated during senescence and is instead lost from the tree (Näsholm, 1994). However, the weak responsiveness of photosynthetic capacity to N reported in conifers (Reich et al., 1995; Han et al., 2004; Wyka et al., 2012; Tarvainen et al., 2013) may in part be explained by N allocation to such non-photosynthetic compounds. Furthermore, if leaf-scale nitrogen-use efficiency (NUE) varies within the canopy in response to patterns in P allocation or arginine synthesis, accurate predictions of the effects of N deposition on stand-scale C cycling may require models to include these factors.

We studied how needle-scale photosynthetic capacity and long-term shoot-scale photosynthetic performance in strongly N-limited *P. sylvestris* trees responded to N fertilization. Furthermore, we assessed the within-canopy N and P allocation patterns under low and high N availabilities, and their potential impacts on canopy-scale C assimilation. We hypothesized that increased N availability would lead to (1) increased shoot-scale photosynthesis, (2) lower shoot-scale NUE, (3) less optimal within-canopy N allocation pattern with respect to maximizing canopy-scale C gain due to N-binding to non-photosynthetic compounds and a greater limitation of photosynthesis by P deficiency in the upper canopy, and further that (4) canopy-scale C uptake may become substantially limited by P and arginine synthesis following N addition on N-poor boreal stands.

## Materials and methods

### Site description

This study was carried out in northern Sweden at Rosinedalsheden experimental forest (64° 10′N, 19° 45′E, 145 m a.s.l.) in August 2013. The experimental site was established for studies of the effects of N addition on stand-scale C cycling under N-poor boreal conditions. The homogeneous *P. sylvestris* forest was regenerated from seed trees in the 1920's and the stand was approximately 90-years-old at the time of the current study. Here we use data from the 15 ha control and high-N plots, one of each, within the experiment. The ambient N deposition rate at the site is < 5 kg N ha^−1^ yr^−1^ (Högberg et al., 2010). The high-N plot was annually fertilized with NH_4_NO_3_ corresponding to 100 kg N ha^−1^ yr^−1^ between 2006 and 2011, and 50 kg N ha^−1^ yr^−1^ from 2012 onwards. The fertilizer also included Ca (5%), Mg (2.4%) and B (0.2%) (Lim et al., 2015). The mean annual temperature and precipitation at Svartberget research station located within 8 km of the studied plots were 1.8°C and 614 mm, respectively, in 1981–2010 (Laudon et al., 2013). The weakly podzolised fine sandy soils have a thin (2–5 cm) organic layer and a C:N ratio of approximately 40 (Hasselquist et al., 2012).

The stand densities at the control and fertilized plots were 1010 ± 125 and 857 ± 83 trees ha^−1^ (mean ± SD) in 2013, respectively. The mean tree height in 2013 was 17.5 ± 0.0 m (mean ± SD) at the control plot compared to 16.6 ± 0.2 m at the fertilized plot, and the leaf area index *c*. 2.7 on the control plot and *c*. 3.1 at the fertilized plot (Lim et al., 2015). Based on dry mass, current-year needles accounted for approximately 29 and 37%, 1-year-old needles 33 and 40%, and 2-year-old needles 24 and 17% of the total needle biomass, at the control and fertilized plots, respectively. The oldest needles at both plots were 4 years old. The stands and the understory are described in more detail in Lim et al. (2015) and Hasselquist et al. (2012), respectively.

### Photosynthetic capacity measurements

Needles were accessed using 16 m tall scaffolding towers located near the centers of the experimental plots. At each plot photosynthetic responses to intercellular CO_2_ concentration (*A*-*C*_i_ curves) were determined at three canopy levels in four dominant and co-dominant trees, growing within 10 m of each other. In the upper canopy measurements were made on current-year, 1-year-old and 2-year-old needles; in the middle and lower canopy only current-year and 1-year-old needles were measured. The *A*-*C*_i_ curves were determined from five attached fascicles (10 needles) using a LI-6400 Portable Photosynthesis System (Li-Cor Biosciences, Lincoln, NE, U.S.A.), with the standard 2 × 3 cm chamber and a light source (6400-02B LED Light Source), running in energy balance mode. To avoid mutual shading, the needles were separated and fixed in a horizontal plane at both ends using black PVC tape prior to insertion in the measurement chamber. Before each individual measurement series leaks were tested for by breathing around the chamber and monitoring the [CO2] inside it. The response of net photosynthesis (*A*_n_) to photosynthetic photon flux density (*Q*) was measured after a period of dark acclimation for *Q* ranging from 0 to 1500 μmol m^−2^ s^−1^ with [CO_2_] set to 400 μmol mol^−1^. The CO_2_ response of *A*_n_ was measured immediately following the light response measurement for a [CO_2_] range from 60 to 2000 μmol mol^−1^ with *Q* set to 1500 μmol m^−2^ s^−1^. The *A*-*C*_i_ and the light response curves were determined at 20°C for all shoots.

Apparent quantum yield (α) was determined using the response of *A*_n_ to *Q* in the 30–75 μmol m^−2^ s^−1^ range. The photosynthetic capacity parameters (the maximum rate of ribulose bisphosphate saturated carboxylation, *V*_cmax_, the maximum photosynthetic electron transport rate, *J*_max_, and the rate of triose phosphate utilization, TPU) were estimated from the *A*-*C*_i_ curves as described in Tarvainen et al. (2013). Briefly, the model by Farquhar et al. (1980), including TPU limitation (as in Sharkey et al., 2007) was modified to estimate the capacity parameters based on *C*_i_ rather than chloroplast CO_2_ concentrations. In all analyses, data points with *C*_i_ below 100 μmol mol^−1^ were assigned *V*_cmax_-limited. For the *J*_max_ calculations, the value of the quantum yield of electron transport was set to 0.3 (Long et al., 1993) and the parameter representing the curvature of the light response to 0.9. The temperature response functions of the photosynthetic CO_2_ compensation point and Michaelis-Menten coefficients of Rubisco were taken from Bernacchi et al. (2001) and the temperature response functions of Bernacchi et al. (2001, 2003) were used to scale the capacity parameters to 25°C in order to facilitate comparison with previously reported values. Because no indication of TPU limitation was found for any of the measured shoots (data not shown), only *V*_cmax_ and *J*_max_ were considered in subsequent analyses of needle photosynthetic capacities and their responses to foliar nutrient contents.

### Continuous gas exchange measurements

Each plot was equipped with a custom-made multi-channel gas exchange system with an infrared gas analyzer (CIRAS-1 PP Systems, Hitchin Herts, UK) running in open mode (Wallin et al., 2001). Shoot-scale CO_2_ and H_2_O exchanges were measured continuously at each plot on two of the trees included in the photosynthetic capacity measurements. The measurements were made on 1-year-old attached shoots located at three canopy levels (in total 12 shoots, six per plot), near the shoots used for the capacity measurements. Shoot segments (55 mm) were inserted in temperature-controlled cuvettes modified for *P. sylvestris* (Image S1). The tubing connecting the cuvettes and the analyzers was insulated and heated. In order to avoid condensation within the cuvettes their temperatures were kept on average 0.2°C above the ambient by Peltier heat exchangers. The shoot-incident *Q* was measured with leveled quantum sensors [PAR-1(M), PP Systems, Hitchin Hertz, UK] placed within 5 cm of the shoot. The cuvettes were constructed from transparent Plexiglas that transmitted *c*. 90% of the incident *Q* (Hall et al., 2013). Shoot-scale *A*_n_ and *C*_i_ were determined using the equations of von Caemmerer and Farquhar (1981). Thus, both estimates were influenced by the respiration of woody twig. Two types of measurement cycles were run during the experiment and each shoot position was measured either for 30 s after a flow stabilization period of 270 s once every 30 min, or for 420 s after a stabilization period of 780 s once every 120 min. At a given time, the cycle times were the same for all cuvettes at one plot, but differed between the plots. The data used in the current study were collected between 1st and 31st of August 2013, and the estimates of mean long-term gas exchange rates were based on days with less than 10% gaps in the daily data set (56% of data).

The continuously measured data were used to calculate the long-term light-use efficiency (LUE) defined as mean *A*_n_ divided by mean *Q*, with the *Q* observation by the cuvette sensor multiplied by 0.9 to account for transmission losses through the cuvette wall. The long-term nitrogen- and phosphorus-use efficiencies (NUE and PUE, respectively) were defined as mean *A*_n_ divided by N or P per projected needle area, *N*_a_ or *P*_a_ (in g nutrient m^−2^ needle) respectively. Thus, LUE is expressed as mol CO_2_ mol^−1^ photons and NUE and PUE as mol CO_2_ g^−1^ N s^−1^ and mol CO_2_ g^−1^ P s^−1^, respectively.

### Determination of needle properties

The needles from the capacity and continuous measurements were harvested in August and November of 2013, respectively. The projected needle areas were determined using a flatbed scanner (Epson 1600+ equipped for dual scanning) and WinSEEDLE Pro 5.1a (Regent Instruments Inc., Quebec City, Canada) analysis software. The needle segments (20 mm) used in capacity measurements were cut along the edges of the tape holding them in place, and the needles used in the continuous measurements were detached from the shoot axis prior to scanning. The needle dry mass was determined after drying to constant weight at 70°C and an elemental analyzer (EA 1108, Fison Instruments, Rodano, Italy) was used to determine the mass-based needle N contents (*N*_m_). Needle total Kjeldahl P contents were determined using Seal AutoAnalyzer HR 3 (Seal Analytical, Nordstedt, Germany; method G-189-97). The foliar nutrient contents of *P. sylvestris* exhibit some seasonality (Tamm, 1955; Näsholm and Ericsson, 1990). Therefore, *N*_m_ and the mass-based needle P contents (*P*_m_) of the continuously measured needles, sampled later in the year, were expected to be greater than those of the needles used in the capacity measurements, although the two sets of needles likely had similar foliar nutrient contents in August. Furthermore, *P. sylvestris* needles tend to rotate around their axis and, thus, using whole needles for determining the leaf mass per projected needle area (*LMA*) results in greater estimates of *LMA*, *N*_a_, and *P*_a_ than analyses using shorter needle segments. This effect was quantified in a separate analysis that showed that *LMA*, *N*_a_, and *P*_a_ estimated for such segments were 12.5 ± 2.6% (*n* = 6) lower than for whole needles (Table S1).

In order to assess the within-canopy and age-related variation in arginine N, and its fraction of total needle N, an additional needle sampling on the studied trees was carried out in October 2014. Needles were collected from positions adjacent to those of the shoots used for capacity measurements the year before and stored at −20°C prior to analysis of their amino acid contents by reverse-phase liquid chromatography using a Waters Ultra High Performance (UPLC) system with a Waters Tunable UV detector (Waters, Corporation, Milford, MA, U.S.A.) following the procedure described in Inselsbacher et al. (2011). Needle N contents excluding arginine N were estimated assuming that the fractions of arginine N of total needle N at the studied canopy levels were equal in 2013 and 2014.

The light climate of each shoot used in the photosynthetic capacity measurements is expressed as diffuse non-interceptance (openness) at the shoot location measured using LAI-2200 Plant Canopy Analyzer (Li-Cor Biosciences, Lincoln, Nebraska). The LAI-2200 was run in two sensor mode with one of the sensors placed above the canopy. The vertical variation in openness is shown in Supplementary material (Figure S1).

### Data analysis and statistics

Because the overall experimental design consisted of non-replicated plots centered on eddy-covariance flux measurement towers and due to the need of scaffolding towers for crown access, the measured trees were not randomly selected. While this raises concerns regarding pseudo-replication (Hurlbert, 1984) in response to potential between plots differences prior to the initial fertilizer application, the aim of the current experiment was to understand the photosynthetic constraints of *P*. *sylvestris* trees with strongly differing foliar N contents growing in the field under otherwise comparable environmental conditions rather than to study treatment effects on the stand-scale *per se*.

All statistical analyses were carried out in IBM SPSS Statistics 20 (IBM Corporation, Armonk, NY, U.S.A.). A three-way repeated measures ANOVA (RMA) with needle age and canopy position as within-subjects factors and fertilizer treatment (plot) as a between-subjects factor was utilized to detect significant differences (*P* < 0.05) in needle photosynthetic, structural and chemical properties. Shapiro-Wilk's and Mauchly's tests were performed to test the data for normality and sphericity, respectively. When the sphericity assumption was met the factor effects were evaluated based on the multivariate tests (Wilks' lambda), and if this assumption was violated the degrees of freedom were corrected using Greenhouse-Geisser estimates of sphericity. In cases where the needle properties of 2-year-old needles were measured only in the upper canopy (see Tables 1, 2), this age class was excluded from the analyses by three-way RMA. Separate tests with two-way RMA, with fertilizer treatment and age as factors, were used to evaluate the effects of needle age, including all three age classes, on the measured needle properties in the upper canopy. In cases where significant interactions between two factors were detected by RMA, tests of simple main effects were carried out to assess the responses to the individual factors. Bonferroni corrections were applied to *post-hoc* analyses of the effects of canopy position and needle age when appropriate.

**Table 1:** **Needle properties in relation to canopy position and age (years) in the ***Pinus sylvestris*** shoots used for photosynthetic capacity measurements at the fertilized (F) and control (C) plots (Mean ± SD, ***n*** = 3–4)**.

		**Age**	**0**		**1**		**2**	
	**Unit**	**Position**	**F**	**C**	**F**	**C**	**F**	**C**
Height	m	Upper	14.3 ± 0.8	14.3 ± 0.6	14.2 ± 0.9	14.3 ± 0.4	14.1 ± 0.9	14.3 ± 0.4
		Mid	12.8 ± 0.6	13.0 ± 0.5	12.8 ± 0.6	12.9 ± 0.5	n.a.	n.a.
		Lower	10.8 ± 0.6	11.1 ± 0.5	10.9 ± 0.5	11.2 ± 0.7	n.a.	n.a.
Openness	Fraction	Upper	0.56 ± 0.12	0.65 ± 0.14	0.50 ± 0.08	0.60 ± 0.05	0.55 ± 0.14	0.62 ± 0.05
		Mid	0.28 ± 0.05	0.45 ± 0.02	0.28 ± 0.08	0.46 ± 0.12	n.a.	n.a.
		Lower	0.20 ± 0.05	0.25 ± 0.05	0.17 ± 0.04	0.22 ± 0.03	n.a.	n.a.
*LMA*	g m^−2^	Upper	248 ± 21	258 ± 13	269 ± 14	257 ± 10	307 ± 26	277 ± 7
		Mid	218 ± 51	230 ± 26	238 ± 16	238 ± 22	n.a.	n.a.
		Lower	207 ± 13	205 ± 9	230 ± 24	213 ± 26	n.a.	n.a.
*N*_a_	g N m^−2^	Upper	4.7 ± 0.8	2.9 ± 0.3	6.5 ± 0.5	3.1 ± 0.6	7.9 ± 0.7	2.9 ± 0.5
		Mid	3.6 ± 0.8	2.6 ± 0.4	5.5 ± 0.2	3.0 ± 0.2	n.a.	n.a.
		Lower	3.7 ± 0.9	2.5 ± 0.4	5.3 ± 1.0	2.8 ± 0.5	n.a.	n.a.
*N*_m_	mg g^−1^	Upper	18.8 ± 1.6	11.3 ± 1.0	24.2 ± 1.7	12.0 ± 2.1	25.8 ± 3.2	10.5 ± 1.9
		Mid	16.8 ± 1.5	11.4 ± 0.7	23.2 ± 0.9	12.7 ± 0.8	n.a.	n.a.
		Lower	17.8 ± 3.1	12.0 ± 1.2	22.9 ± 2.1	13.0 ± 0.9	n.a.	n.a.
*P*_a_	g P m^−2^	Upper	0.48 ± 0.19	0.35 ± 0.07	0.39 ± 0.14	0.31 ± 0.10	0.47 ± 0.07	0.30 ± 0.03
		Mid	0.37 ± 0.19	0.30 ± 0.06	0.31 ± 0.02	0.26 ± 0.03	n.a.	n.a.
		Lower	0.37 ± 0.05	0.29 ± 0.02	0.40 ± 0.17	0.26 ± 0.03	n.a.	n.a.
*P*_m_	mg g^−1^	Upper	1.9 ± 0.7	1.4 ± 0.2	1.4 ± 0.6	1.2 ± 0.4	1.5 ± 0.3	1.1 ± 0.1
		Mid	1.6 ± 0.4	1.3 ± 0.1	1.3 ± 0.0	1.1 ± 0.1	n.a.	n.a.
		Lower	1.8 ± 0.2	1.4 ± 0.0	1.7 ± 0.5	1.2 ± 0.1	n.a.	n.a.
*P*:N	g P g^−1^ N	Upper	0.10 ± 0.03	0.12 ± 0.01	0.06 ± 0.02	0.10 ± 0.02	0.06 ± 0.01	0.11 ± 0.01
		Mid	0.10 ± 0.03	0.11 ± 0.01	0.06 ± 0.00	0.09 ± 0.00	n.a.	n.a.
		Lower	0.10 ± 0.01	0.12 ± 0.01	0.07 ± 0.02	0.09 ± 0.01	n.a.	n.a.
Total AA	mg N g^−1^	Upper	1.54 ± 0.20	0.010 ± 0.004	2.03 ± 0.53	0.009 ± 0.003	2.22 ± 0.71	0.012 ± 0.002
		Mid	1.55 ± 0.53	0.008 ± 0.002	1.81 ± 0.66	0.011 ± 0.004	1.83 ± 0.52	0.027 ± 0.021
		Lower	1.21 ± 0.87	0.014 ± 0.005	1.31 ± 1.03	0.015 ± 0.007	1.52 ± 0.90	0.012 ± 0.006
ARG	mg N g^−1^	Upper	1.45 ± 0.19	0.002 ± 0.001	1.95 ± 0.49	0.002 ± 0.000	2.14 ± 0.68	0.002 ± 0.001
		Mid	1.44 ± 0.48	0.002 ± 0.000	1.73 ± 0.62	0.002 ± 0.001	1.76 ± 0.49	0.002 ± 0.000
		Lower	1.12 ± 0.80	0.003 ± 0.001	1.24 ± 0.98	0.004 ± 0.002	1.45 ± 0.85	0.003 ± 0.002
ARG % of AA	%	Upper	94.5 ± 0.5	17.3 ± 0.2	95.9 ± 1.9	18.8 ± 0.2	96.5 ± 2.4	14.3 ± 0.1
		Mid	93.6 ± 2.8	18.4 ± 0.1	95.5 ± 2.9	20.1 ± 0.2	96.3 ± 2.3	11.2 ± 2.1
		Lower	92.3 ± 4.6	19.7 ± 0.2	93.8 ± 3.9	28.9 ± 0.3	95.0 ± 3.4	20.0 ± 0.3
ARG % of *N*_m_	%	Upper	7.9 ± 1.3	0.015 ± 0.008	9.5 ± 2.4	0.013 ± 0.004	n.a.	n.a.
		Mid	7.6 ± 2.4	0.013 ± 0.002	8.6 ± 3.0	0.019 ± 0.007	n.a.	n.a.
		Lower	6.1 ± 4.5	0.022 ± 0.010	5.9 ± 3.9	0.044 ± 0.020	n.a.	n.a.

*The between plots and within-canopies variations were analyzed using repeated measures ANOVA, the statistics are presented in **Table 3** and Table S2 (for age-related variation in the upper canopy). Data collected in August 2013: Height; Openness; LMA, leaf mass per unit area; N_a_ and N_m_, needle nitrogen content per unit area and mass, respectively; P_a_ and P_m_, needle phosphorus content per unit area and mass, respectively; P:N, ratio between needle phosphorus and nitrogen contents. Data collected in October 2014: Total AA, total free amino acid N content; ARG, arginine N content; n.a., not available*.

**Table 2:** **Needle photosynthetic capacity in relation to canopy position and age (years) in the ***Pinus sylvestris*** shoots used for photosynthetic capacity measurements at the fertilized (F) and control (C) plots in August 2013 (Mean ± SD, ***n*** = 3–4)**.

		**Age**	**0**		**1**		**2**	
	**Unit**	**Position**	**F**	**C**	**F**	**C**	**F**	**C**
α	μmol μmol^−1^	Upper	0.048 ± 0.006	0.062 ± 0.012	0.044 ± 0.012	0.054 ± 0.012	0.057 ± 0.006	0.048 ± 0.010
		Mid	0.048 ± 0.014	0.044 ± 0.013	0.050 ± 0.013	0.048 ± 0.008		
		Lower	0.049 ± 0.023	0.052 ± 0.011	0.067 ± 0.013	0.056 ± 0.009		
*V*_cmax_	μmol m^−2^ s^−1^	Upper	99 ± 6	94 ± 22	119 ± 5	99 ± 12	96 ± 11	86 ± 11
		Mid	111 ± 11	87 ± 9	118 ± 15	93 ± 7		
		Lower	108 ± 14	88 ± 17	120 ± 24	92 ± 6		
*J*_max_	μmol m^−2^ s^−1^	Upper	176 ± 14	170 ± 38	184 ± 13	167 ± 23	153 ± 22	151 ± 32
		Mid	163 ± 20	146 ± 15	184 ± 20	157 ± 7		
		Lower	174 ± 12	166 ± 18	211 ± 51	162 ± 17		
*J*_max_:*V*_cmax_	ratio	Upper	1.8 ± 0.1	1.8 ± 0.1	1.5 ± 0.1	1.7 ± 0.2	1.6 ± 0.1	1.7 ± 0.2
		Mid	1.5 ± 0.0	1.7 ± 0.0	1.6 ± 0.2	1.7 ± 0.1		
		Lower	1.6 ± 0.1	1.9 ± 0.3	1.8 ± 0.1	1.8 ± 0.2		
*V*_cmax_:N	μmol g^−1^ N s^−1^	Upper	21.7 ± 5.3	32.7 ± 8.7	18.4 ± 2.0	32.6 ± 4.5	11.8 ± 2.1	29.9 ± 4.3
		Mid	31.2 ± 4.6	33.9 ± 8.1	21.3 ± 2.1	30.9 ± 4.5		
		Lower	30.7 ± 8.5	35.7 ± 5.3	22.9 ± 4.9	33.6 ± 4.2		
*J*_max_:N	μmol g^−1^ N s^−1^	Upper	37.9 ± 9.4	59.1 ± 14.6	28.5 ± 4.3,	54.7 ± 6.7	19.0 ± 4.7	51.6 ± 5.5
		Mid	45.6 ± 6.4	57.0 ± 13.9	33.4 ± 3.3a	52.0 ± 4.3		
		Lower	50.1 ± 12.2	67.6 ± 2.6	40.2 ± 9.9	58.9 ± 7.1		
*V*_cmax_:P	μmol g^−1^ P s^−1^	Upper	243 ± 145	277 ± 86	334 ± 102	337 ± 89	200 ± 38	287 ± 54
		Mid	342 ± 110	303 ± 92	382 ± 32	361 ± 70		
		Lower	296 ± 46	303 ± 54	328 ± 102	356 ± 30		
*J*_max_:P	μmol g^−1^ P s^−1^	Upper	424 ± 254	501 ± 147	523 ± 180	567 ± 151	320 ± 52	498 ± 101
		Mid	498 ± 155	511 ± 159	599 ± 69	605 ± 79		
		Lower	486 ± 69	572 ± 35	573 ± 182	626 ± 72		

*The between plots and within-canopies variations were analyzed using repeated measures ANOVA, the statistics are presented in **Table 3** and Table S2 (for age-related variation in the upper canopy). α, apparent quantum yield within a photosynthetic photon flux density range of 30–75 μmol m^−2^ s^−1^ at 20°C; V_cmax_ and J_max_, the maximum rates of ribulose bisphosphate saturated carboxylation and photosynthetic electron transport scaled to 25°C, respectively; N and P, needle nitrogen and phosphorus content, respectively*.

The photosynthetic capacity responses to needle nutrient contents were evaluated by regression analyses based on: (i)*N*_a_, (ii)*P*_a_, (iii)*N*_a_ and *P*_a_, (iv) area-based needle N content excluding arginine N (*N*_ax_), and (v)*N*_ax_ and *P*_a_. When both N and P were included (i.e., cases iii and v), the co-dependencies of N and P were determined by multiple linear regression analysis that also included the N^*^P interaction term. Because fertilizer application was expected to affect the relationships between N, P and photosynthetic capacity parameters, the multiple regression analyses were carried out separately for each plot. Regression analyses were also used to investigate the between plots differences in the long-term shoot-scale photosynthetic performance responses to environmental variables and needle properties. The differences between the regression slopes and intercepts were evaluated using ANCOVA. If the relationship between the dependent and independent variables was non-linear data were log-log transformed prior to the ANCOVA analysis.

### Modeling of the long-term photosynthetic performance

The shoot-scale *A*_n_ was modeled based on the approach by Farquhar et al. (1980) as described in e.g., Harley et al. (1992), Medlyn et al. (2002), and Walker et al. (2014). According to this approach *A*_n_ can be estimated based on the minimum of the two rates, Rubisco activity (*W*_c_) and Ribulose-1,5-bisphosphate (RuBP) regeneration (*W*_j_), limiting the gross carboxylation rate, the CO_2_ compensation point (Γ^*^), *C*_i_ and mitochondrial respiration (*R*_d_):

(1) $$\begin{array}{l} A_{n}=\text{min}\left\{W_{c,}W_{j}\right\}\left(1-\frac{\Gamma^{*}}{C_{i}}\right)-R_{d} \end{array}$$

The Rubisco-limited gross carboxylation rate, *W*_c_, is described as:

(2) $$\begin{array}{l} W_{c}=V_{cmax}\frac{C_{i}}{C_{i}+K_{c}\left(1+\frac{O_{i}}{K_{o}}\right)} \end{array}$$

where *K*_c_ and *K*_o_ are the Michelis-Menten coefficients of Rubisco for CO_2_ and O_2_, respectively, and *O*_i_ is the intercellular O_2_ concentration. The RuBP regeneration-limited gross carboxylation rate, *W*_j_, is described as:

(3) $$\begin{array}{l} W_{j}=\frac{J}{4}\times \frac{C_{i}}{C_{i}+2\Gamma^{*}} \end{array}$$

where J is the rate of electron transport that is related to *Q*:

(4) $$\begin{array}{l} J=\frac{\alpha_{e}Q}{{\left(1+{\left(\frac{\alpha_{e}Q}{J_{max}}\right)}^{2}\right)}^{0.5}} \end{array}$$

where α_e_ is the apparent quantum yield of electron transport, assumed to be 0.24 mol electrons mol^−1^ photons (Harley et al., 1992).

The model was initially parameterized for 1-year-old needles utilizing plot specific data to test if model predictions of the long-term *A*_n_ were reasonable compared to the observations. Because no significant within-canopy capacity differences were detected (Tables 2, 3), canopy averages of *V*_cmax_ and *J*_max_ were used. Dark respiration (*R*_d_), at 0°C, and its temperature response were determined from the continuous shoot-scale measurements made when *Q* was below 2 μmol m^−2^ s^−1^. The *R*_d_ value, thus, includes the respiration from the woody twig but does not account for the reduction in respiration rates in light (e.g., Way and Yamori, 2014). The model was driven with needle temperature, *Q* and *C*_i_ data from the continuous measurements. As for the LUE calculation, the observed incident *Q* value was multiplied by 0.9 to account for the loss of light due to transmission through the cuvette walls. Occasionally condensation, and subsequent evaporation, of water in the tubing caused the *C*_i_ estimates to be in error; therefore data was excluded from the modeling when the *C*_i_:*C*_a_ ratio was < 0.2 or > 1.2 (*c*. 10% of the data removed). These cut-off *C*_i_:*C*_a_ ratios were determined from the LI-6400 derived light response curves, with the lower boundary corresponding to the minimum *C*_i_:*C*_a_ ratio at light saturation and the upper boundary to the maximum *C*_i_:*C*_a_ ratio in darkness.

**Table 3:** **Repeated measures ANOVA statistics for between plots and within-canopies variation in the needle properties of current-year and 1-year-old shoots presented in Tables 1, 2**.

**Variable**	**Position**	**Age**	**Plot**	Position^*^Age	Position^*^Plot	Age^*^Plot	Position^*^Age^*^Plot
Height	<0.001	n.s.	n.s.	n.s.	n.s.	n.s.	n.s.
Openness	<0.001 (C)	0.004	0.023 (U)	n.s.	0.044	n.s.	n.s.
	≤0.038 (F)		<0.001 (M)				
			n.s. (L)				
*LMA*	<0.001	n.s.	n.s.	n.s.	n.s.	n.s.	n.s.
*N*_a_	0.029	n.s. (C) <0.001 (F)	< 0.001	n.s.	n.s.	0.010	n.s.
*N*_m_	n.s.	n.s. (C) < 0.001 (F)	<0.001	n.s.	n.s.	0.005	n.s.
*P*_a_	n.s.	n.s.	n.s.	n.s.	n.s.	n.s.	n.s.
*P*_m_	0.039	0.036	0.026	n.s.	n.s.	n.s.	n.s.
*P*:N	n.s.	<0.001	0.009	n.s.	n.s.	n.s.	n.s.
Total AA^*^	n.s.	n.s.	0.001	n.s.	n.s.	n.s.	n.s.
ARG^*^	n.s.	n.s.	0.001	n.s.	n.s.	n.s.	n.s.
ARG % of AA^*^	n.s.	<0.001 (C)0.38 (F)	<0.001	n.s.	0.001	n.s.	n.s.
ARG % of *N*_m_^*^	n.s.	n.s.	0.005	n.s.	n.s.	n.s.	n.s.
α	0.040	n.s.	n.s.	n.s.	n.s.	n.s.	n.s.
*V*_cmax_	n.s.	n.s.	0.004	n.s.	n.s.	n.s.	n.s.
*J*_max_	n.s.	n.s.	0.049	n.s.	n.s.	n.s.	n.s.
*J*_max_:*V*_cmax_	0.008 (0) n.s. (1)	0.036 (U) n.s (M) n.s. (L)	0.008	0.047	n.s.	n.s.	n.s.
*V*_cmax_:N	n.s.	n.s.	0.017	n.s.	n.s.	n.s.	n.s.
*J*_max_:N	n.s.	n.s.	0.003	n.s.	n.s.	n.s.	n.s.
*V*_cmax_:P	n.s.	n.s.	n.s.	n.s.	n.s.	n.s.	n.s.
*J*_max_:P	n.s.	n.s.	n.s.	n.s.	n.s.	n.s.	n.s.

C, control plot; F, fertilized plot; U, M and L, upper, mid, and lower canopy; 0 and 1, needle age in years. Simple main effects were tested for where significant interactions were detected and Bonferroni corrections were applied for analyses of multiple correlations, n.s., not significant (P > 0.05). For variables marked with

* *data from all three age classes were included in the analyses*.

**Figure 1:**
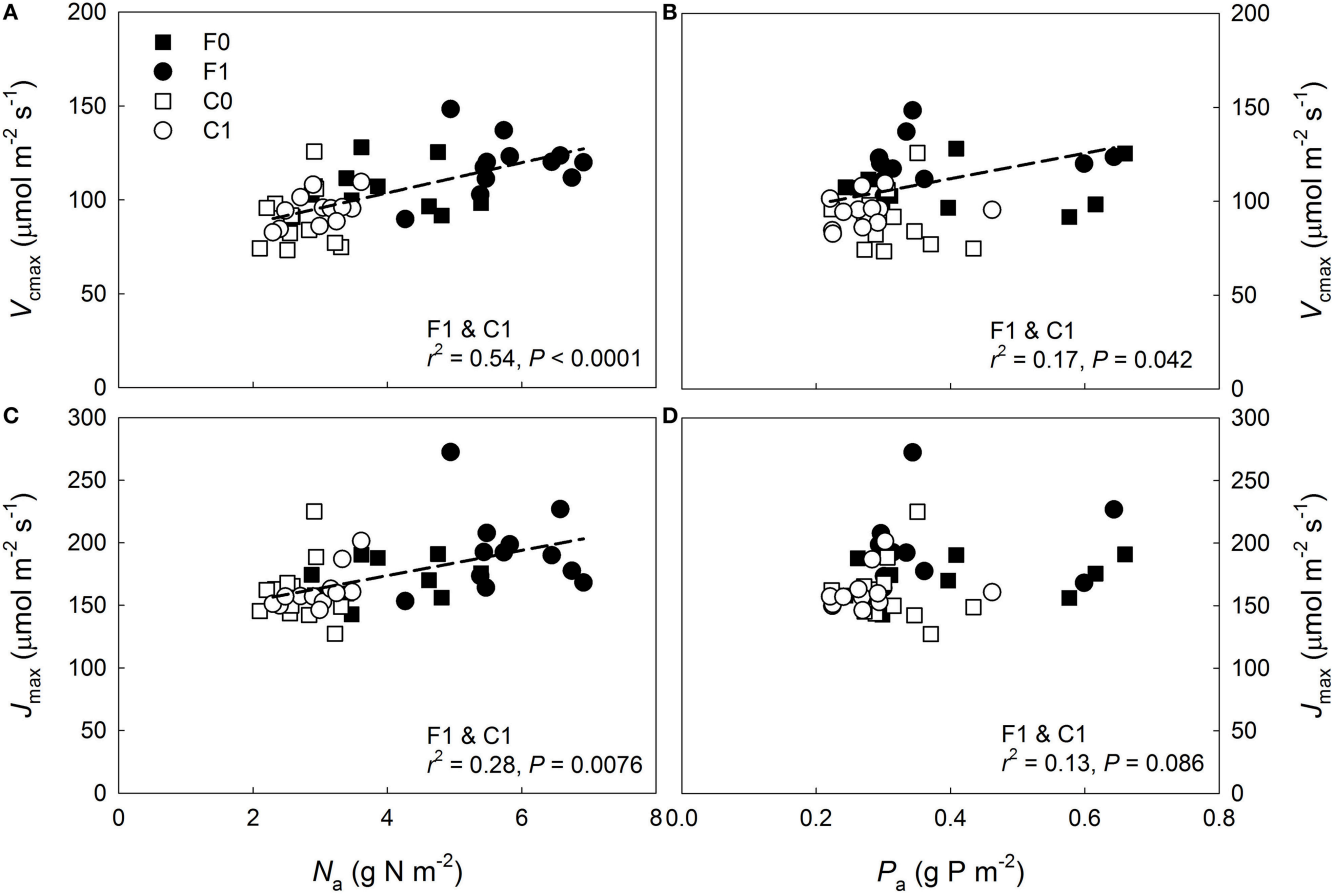
**Photosynthetic capacity vs. needle nitrogen and phosphorus content per surface area (***N***_**a**_ and ***P***_**a**_) in ***Pinus sylvestris*****. Variation in **(A)** maximum carboxylation rate (*V*_cmax_) at 25°C with *N*_a_, **(B)** *V*_cmax_ at 25°C with *P*_a_, **(C)** maximum electron transport rate (*J*_max_) at 25°C with *N*_a_, and **(D)** *J*_max_ at 25°C with *P*_a_. Control plot (C) = open symbols, fertilized plot (F) = filled symbols, squares = current-year needles, circles and dashed lines = 1-year-old needles.

To determine the potential limitation of long-term *A*_n_ by foliar P deficiency and N binding to arginine, the model was run for the two continuously measured upper canopy shoots at the fertilized plot. In the first run the upper canopy mean *V*_cmax_ and *J*_max_ from the capacity measurements on fertilized needles were used together with the continuously observed needle temperature, *Q* and *C*_i_ data. In the second run *V*_cmax_ and *J*_max_ were estimated by extrapolating the *N*_a_ dependencies of *V*_cmax_ and *J*_max_ observed at the control plot (Figures 1A,C) to the mean *N*_a_ of the fertilized upper canopy needles used for the capacity measurements, 6.5 g N m^−2^. Finally, the model was also run with *V*_cmax_ and *J*_max_ estimated specifically for the two continuously measured fertilized upper canopy shoots, both with 7.6 g N m^−2^ when harvested in November.

## Results

### Variation in needle properties in response to nitrogen addition, canopy position, and age

The observations of the light climate (openness) of the shoots used in the photosynthetic capacity measurements suggest that the upper canopy needles received on average at least three times as much diffuse light than the lower canopy needles (Table 1). The continuous measurements, which integrated diffuse and direct light, showed that light availability varied considerably among the canopy positions with the average daily integrated *Q* ranging from 5.2 mol m^−2^ d^−1^ to 23.4 mol m^−2^ d^−1^ at the control plot and from 2.9 mol m^−2^ d^−1^ to 30.0 mol m^−2^ d^−1^ at the fertilized plot (**Figure 3**, x-axes). Trees growing at the fertilized plot exhibited significantly greater *N*_a_ and *N*_m_, as expected, but also greater *P*_m_ and lower P:N ratios than the trees at the control plot, while *P*_a_ did not vary significantly between the plots (Tables 1, 3). The RMA analysis indicated canopy position to be a significant main effect for *LMA*, *N*_a_ and *P*_m_ with *LMA* and *N*_a_ increasing from the lower to the upper canopy. The 1-year-old needles had greater *N*_a_ and *N*_m_, but lower *P*_m_ and P:N ratios than the current-year needles (Tables 1, 3). Significant age^*^plot interactions were found for *N*_a_ and *N*_m_, reflecting the lower needle N content in current-year needles compared to the 1-year-old needles at the fertilized plot (Tables 2, 3). Furthermore, significantly more of the total needle N, 6–10%, was bound to free amino acids, mostly arginine, at the fertilized plot, while < 0.05% of the total needle N was in free amino acids at the control plot (Tables 1, 3).

Apparent quantum yield (α) of the needles did not vary significantly with N availability or age, but was higher in lower than mid-canopy (Tables 2, 3). Both *V*_cmax_ and *J*_max_ were significantly higher at the fertilized than at the control plot. Furthermore, no significant age-related or vertical within-canopy variations in *V*_cmax_ or *J*_max_ were detected for the current-year and 1-year-old needles (Tables 2, 3). However, in the upper canopy the 1-year-old needles had significantly higher *V*_cmax_ than the 2-year-old needles (Table S2). The *J*_max_: *V*_cmax_ ratios were higher at the control plot and also exhibited a significant interaction between needle age and canopy position with low values in the current-year mid-canopy needles (Tables 2, 3).

### Photosynthetic capacity responses to nitrogen, phosphorus, and temperature

When data from both plots were pooled a significant increase in *V*_cmax_ with both *N*_a_ and *P*_a_ was detected for the 1-year-old needles (Figures 1A,B), while the *J*_max_ of the 1-year-old needles increased significantly with *N*_a_ but not with *P*_a_ (Figures 1C,D). The relationships between capacity parameters and *N*_a_ or *P*_a_ were not significant for the current-year needles at either plot (Figures 1A–D, *P* ≥0.13). When data was divided into groups based on plot and age, no significant relationships were found between the capacity parameters and *P*_a_ (Figures 1B,D, *P* = 0.37). The *J*_max_ of 1-year-old needles increased significantly with *N*_a_ at the control plot (*r*^2^ = 0.44, *P* = 0.02) but not at the fertilized plot (*r*^2^ < 0.01, *P* = 0.89), while *V*_cmax_ of the 1-year-old needles did not vary significantly with *N*_a_ at either plot (fertilized plot: *r*^2^ = 0.04, *P* = 0.52, control plot: *r*^2^ = 0.25, *P* = 0.10). Expressing needle N content without arginine bound N did not improve the linear regression fits (*P* = 0.91, data not shown). Both *V*_cmax_:N and *J*_max_:N were significantly greater at the control plot, while rate of return for P investment did not vary significantly between the plots (Tables 2, 3). The relationships between photosynthetic capacity parameters and foliar N or P contents were not affected by needle age or canopy position (Tables 2, 3).

**Figure 2:**
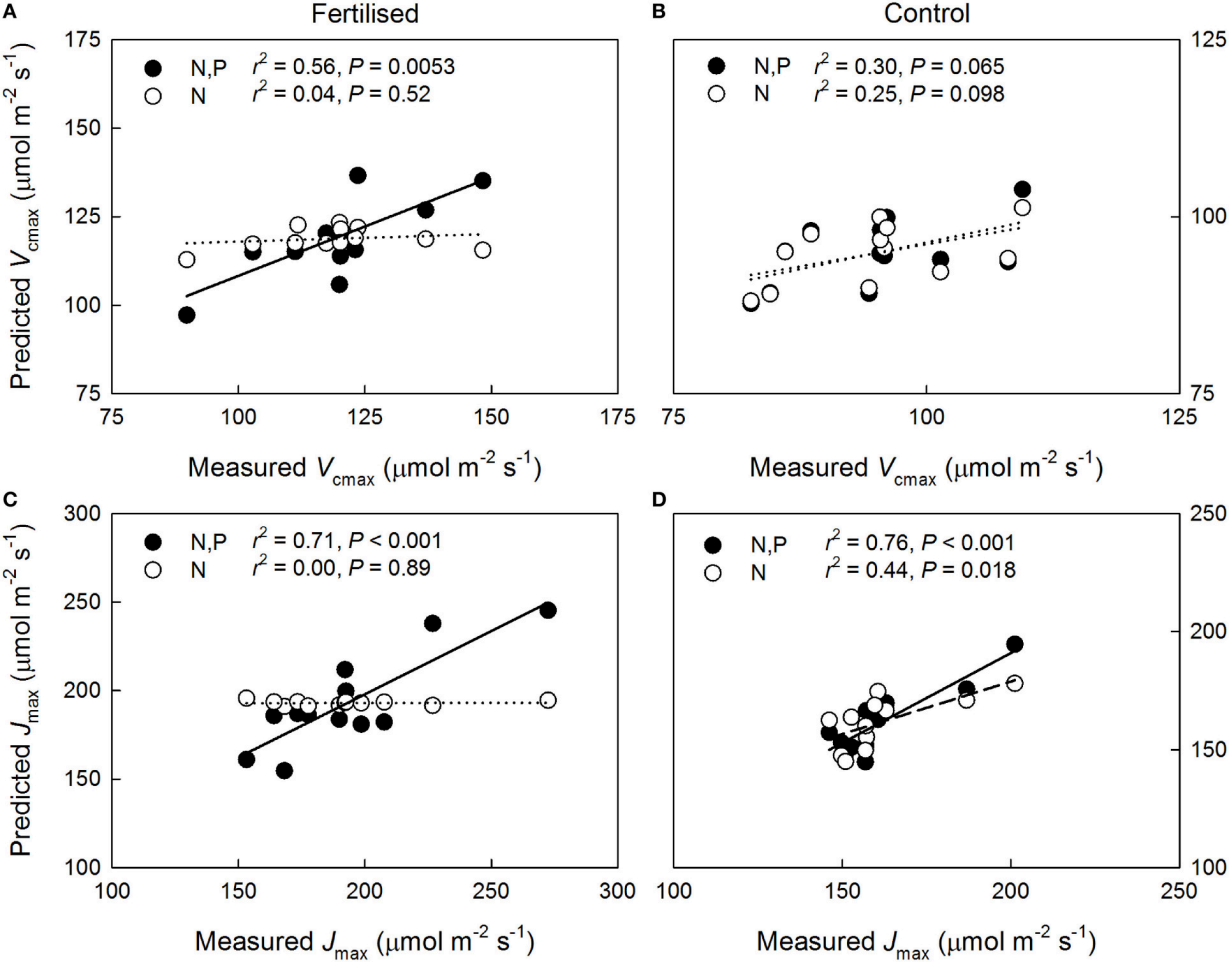
**Relationship between predicted and measured maximum carboxylation rate (***V***_**cmax**_) and maximum electron transport rate (***J***_**max**_) at 25°C in 1-year-old ***Pinus sylvestris*** needles based on (i) needle nitrogen content per unit area, ***N***_**a**_, (N: open circles, dashed lines) and (ii) ***N***_**a**_ and needle phosphorus content per unit area, ***P***_**a**_, (N, P: filled circles, solid lines)**. Sub-figures: *V*_cmax_ on the **(A)** fertilized and **(B)** control plots; *J*_max_ on the **(C)** fertilized, and **(D)** control plots. Dotted lines, non-significant relationships.

The predictions of the *V*_cmax_ of 1-year-old shoots improved following the inclusion of the P and the N^*^P parameters at the fertilized plot (Figure 2A) but not at the control plot (Figure 2B). The importance of including P and N^*^P in *J*_max_ predictions was especially clear at the fertilized plot where the prediction based on *N*_a_ alone was unable to explain the observed variation, whereas including P and N^*^P in the regression model resulted in a strong correlation between the measured and the modeled values (Figure 2C). At the control plot, despite *J*_max_ being significantly related to *N*_a_ alone, including P and N^*^P also improved the *J*_max_ predictions (Figure 2D). The accuracy of the capacity predictions for the current-year needles was unaffected by the inclusion of P and N^*^P for both age classes and plots (Figure S2). Although using *N*_ax_ rather than *N*_a_ in the multiple regression analyses increased the *r*^2^ value of the correlation between the modeled and the measured *V*_cmax_ of the 1-year-old needles at the fertilized plot from 0.56 to 0.77, no statistically significant improvements in the capacity predictions were detected at either plot or for either age class when N was expressed as *N*_ax_ instead of *N*_a_ (P = 0.31, data not shown).

### Long-term shoot-scale carbon uptake

The vertical variation in the needle properties is described with respect to daily mean shoot-incident *Q* measured in August 2013 (Table 4), because the small sample size (*n* = 2–3) precluded using analysis of variance (Table S1). Leaf mass per area and *N*_a_ increased significantly with *Q* at both plots whereas significant *P*_a_ increase was detected only at the fertilized plot. When nutrient contents were expressed on per mass basis they did not vary significantly with light availability at either plot.

**Table 4:** **Needle properties in relation to mean shoot-incident photosynthetic photon flux density (***Q***, μmol m^**−2**^ d^**−1**^) in 1-year-old ***Pinus sylvestris*** shoots used in continuous shoot-scale net gas exchange measurements at the fertilized (F) and control (C) plots in August 2013**.

**Variable**	**Unit**	**Plot**	**Equation**	***r*^2^**	***P*-value**	***n***
*LMA*	g m^−2^	F	4.5x + 239.2	0.87	0.007^*^	6
		C	4.1x + 198.0	0.65	0.029^*^	7
*N*_a_	g N m^−2^	F	0.09x + 5.2	0.88	0.006^*^	6
		C	0.05x + 2.1	0.77	0.010^*^	7
*N*_m_	mg g^−1^	F	−0.01x + 21.6	0.02	0.82	6
		C	0.03x + 10.8	0.09	0.52	7
*P*_a_	g P m^−2^	F	0.006x + 0.5	0.89	0.005^*^	6
		C	0.007x + 0.3	0.52	0.10	6
*P*_m_	mg g^−1^	F	−0.009x + 2.1	0.44	0.15	6
		C	0.003x + 1.5	0.03	0.75	6

LMA, leaf mass per unit area; N_a_ and N_m_, needle nitrogen content per unit area and mass, respectively; P_a_ and P_m_, needle phosphorus content per unit area and mass, respectively. Linear regression equations and statistics are shown (n, number of shoots). Significant vertical gradients are denoted by

* *. Vertical within-canopy variation in Q is shown in Table S1*.

**Figure 3:**
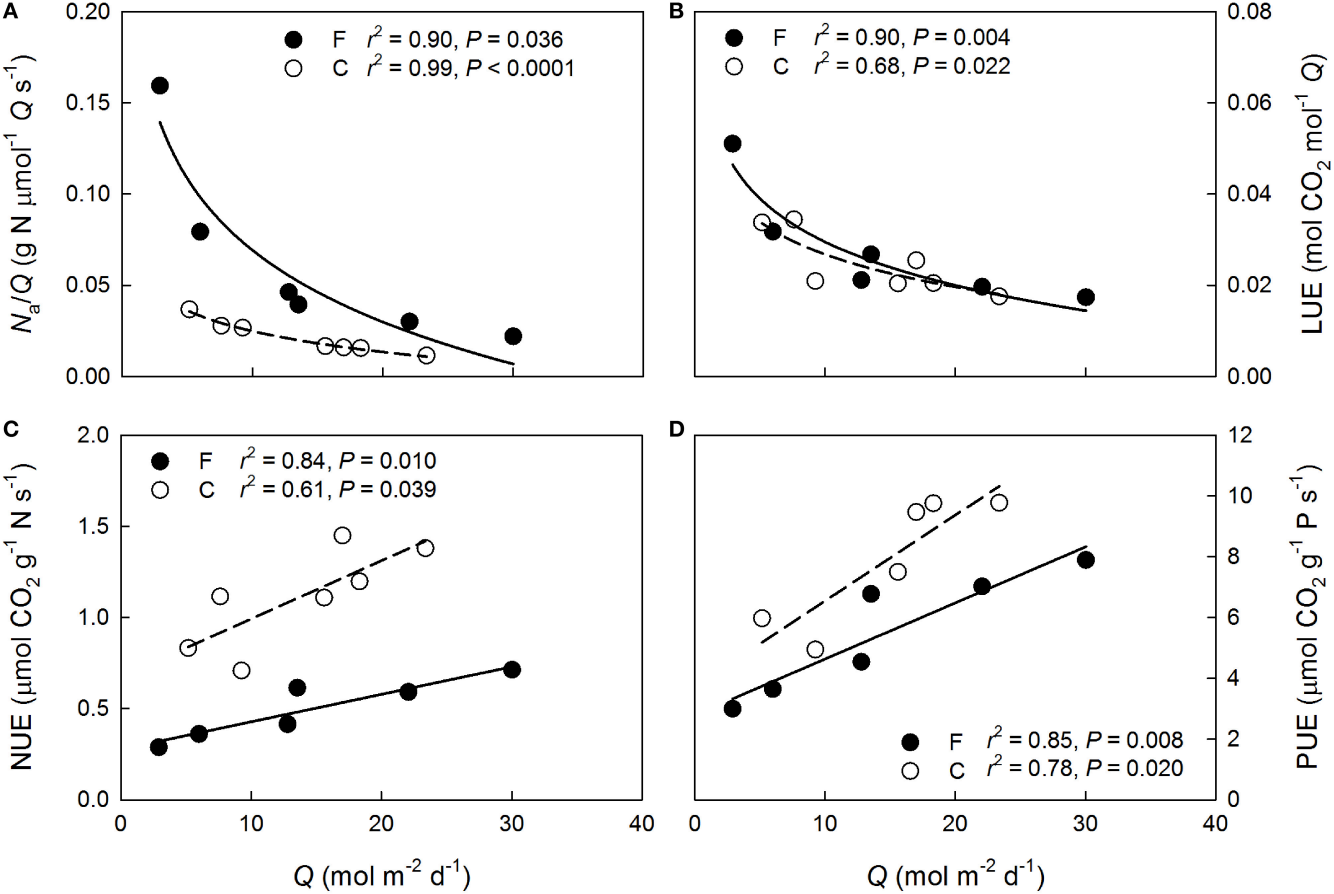
**Variation with daily mean shoot-incident photosynthetic photon flux density (***Q***) in (A) needle N allocation per long-term mean incident light intensity (***N***_**a**_/***Q***), (B) light-use efficiency (LUE), (C) nitrogen-use efficiency (NUE), and (D) phosphorus-use efficiency (PUE) of 1-year-old ***Pinus sylvestris*** shoots within the fertilized (filled circles, solid lines) and the control (open circles, dashed lines) canopies in August 2013**.

**Figure 4:**
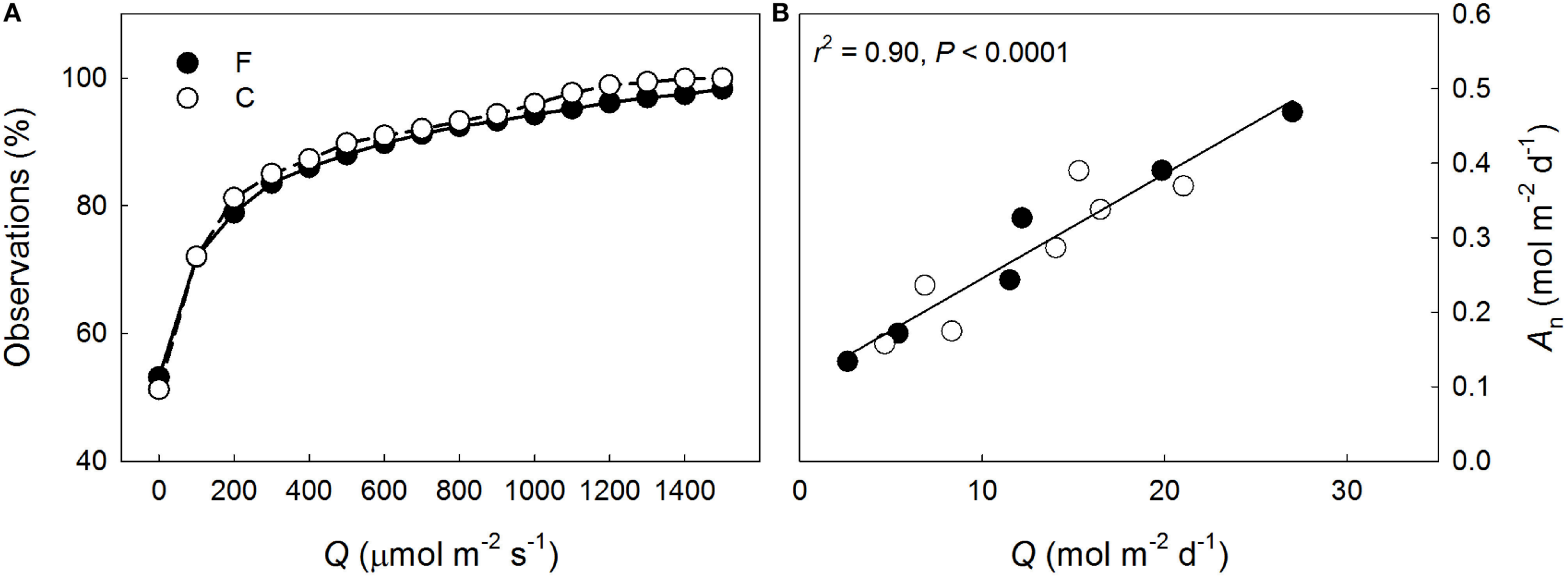
**(A)** Cumulative percentage of incident photosynthetic photon flux density (*Q*) measured adjacent to the continuously measured *Pinus sylvestris* shoots at the fertilized (F, filled symbols) and the control (C, open symbols) plots, and **(B)** the daily mean integrated shoot-scale net photosynthetic performance (*A*_n_) in relation to daily mean *Q* at F and C observed in August 2013. The regression line and statistics in **(B)** are shown for the pooled data.

Shoot-incident *Q* declined more rapidly than *N*_a_ through the canopy at both plots (Figure 3A). Overall more N was allocated to the needles per intercepted *Q* at the fertilized plot (*P* < 0.001) but the within-canopy variation was similar between the plots (*P* = 0.16). Shoot-scale LUE increased non-linearly with decreasing mean *Q* at both plots (Figure 3B). No significant between plots differences were detected in the LUE responses to *Q* (*P* = 0.63), or in LUE in general (*P* = 0.72), indicating that the long-term needle area-based photosynthetic performance between the plots was similar when light availability was accounted for. Because the light availability also was similar between the plots (Figure 4A), the long-term shoot-scale mean daily integrated *A*_n_ did not differ significantly between the plots (Figure 4B, *P* = 0.96). Moreover, the daily integrated *A*_n_ scaled significantly (Figure 4B) and similarly (*P* = 0.96) with daily integrated *Q* at both plots. Nitrogen-use efficiency increased significantly with light availability at both plots (Figure 3C) and the slopes of the NUE responses to *Q* were similar between the plots (*P* = 0.17). Furthermore, the non-significant difference in the regression intercepts indicated that there was no treatment effect on shoot-scale NUE (*P* = 0.052). Like NUE, PUE increased significantly with mean *Q* at both plots (Figure 3D). No significant differences were detected for the intercepts or the slopes of the regressions suggesting that N-addition did not affect either the overall shoot-scale PUE (*P* = 0.26) or the PUE responses to *Q* (*P* = 0.50).

### Limitation of long-term photosynthetic performance

**Figure 5:**
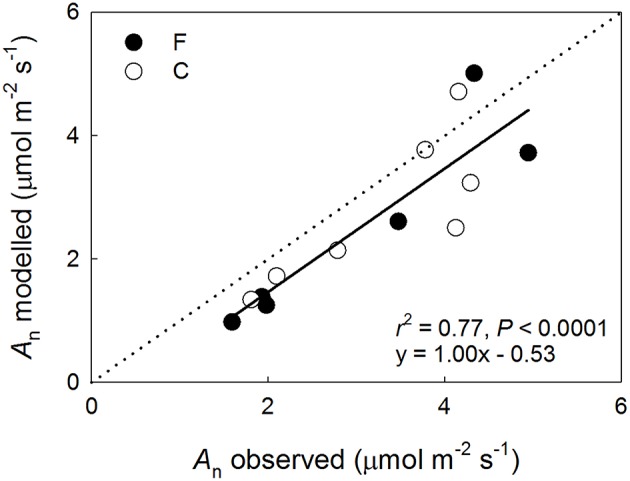
**Modeled vs. observed net photosynthetic performance (***A***_**n**_) of ***Pinus sylvestris*** shoots at the fertilized (F, filled symbols) and the control (C, open symbols) plots in August 2013**. The regression line and statistics are shown for the pooled data.

The data used in the performance modeling are summarized in Table 5. While the model generally underestimated the observed long-term shoot-scale *A*_n_, it performed qualitatively well exhibiting a slope of 1.0 (for data pooled from both plots) when canopy average *V*_cmax_, *J*_max_ and shoot specific environmental drivers were used (Figure 5). Notably, there was a tendency toward a greater variance in the model performance in the upper canopy shoots with the model producing overestimates in some cases.

**Table 5:** **Parameters used for modeling the per unit leaf area needle photosynthetic performance of ***Pinus sylvestris*** on the fertilized (F) and control (C) plots**.

		**Plot average**		**Upper canopy**		
**Parameter**	**Unit**	**F**	**C**	***F*_top_**	***F*_mod_, *N*_a_ = 6.5**	***F*_mod_, *N*_a_ = 7.6**
*V*_cmax_	μmol m^−2^ s^−1^	118	95	119	130	142
*J*_max_	μmol m^−2^ s^−1^	193	162	184	251	279
*R*_d_	μmol m^−2^ s^−1^	0.90	0.92	0.90	0.90	0.90
*Q*_10_	Unitless	2.67	2.44	2.67	2.67	2.67

*V_cmax_ and J_max_, the maximum rates of ribulose bisphosphate saturated carboxylation and photosynthetic electron transport scaled to 25°C, respectively; R_d_, shoot dark respiration at 20°C; Q_10_, relative change in R_d_ to 10°C change in temperature*.

When the model was parameterized specifically for upper canopy shoots at the fertilized plot, the predictions were also reasonable being *c*. 7% lower than the observations (0.37 ± 0.08 mol CO_2_ m^−2^ d^−1^ vs. 0.40 ± 0.04 mol CO_2_ m^−2^ d^−1^ [mean ± SD], respectively; *n* = 2 for both estimates). The second model run estimated the potential limitation of upper canopy long-term *A*_n_ by P deficiency and N allocation to arginine. The non-limited capacity estimates were obtained by extrapolating the capacity-*N*_a_ relationship observed at the control plot (Figure 1) to the mean *N*_a_ observed in the needles used in the capacity measurements, 6.5 g N m^−2^ s^−1^ (Table 1). Based on these capacity estimates and the continuously observed *Q*, needle temperature and *C*_i_, the model predicted a non-limited *A*_n_ of 0.41 ± 0.09 mol CO_2_ m^−2^ d^−1^ (mean ± SD), suggesting a *c*. 10% mean reduction in long-term *A*_n_ for the two shoots due to capacity limitations. When the model was run using capacity estimates based on the observed upper canopy *N*_a_ from the November harvest of the long-term measured shoots, 7.6 g N m^−2^ s^−1^, the predicted *A*_n_ was 0.43 ± 0.09 mol CO_2_ m^−2^ d^−1^, indicating a potential performance limitation of *c*. 13.5%.

## Discussion

Boreal conifers are commonly considered N-limited (e.g., Tamm, 1991). Therefore, it would be reasonable to expect significant shoot-scale photosynthetic performance responses when N is added to such stands. The results of the current study, however, suggest that the aboveground growth response following N fertilization of mature stands (Tamm, 1956; Brix, 1971, 1983; Linder and Axelsson, 1982; Axelsson and Axelsson, 1986; Linder, 1987; Lim et al., 2015) reflects changes in C allocation patterns rather than any enhancements in long-term C uptake per unit leaf area. Furthermore, the reduced C allocation to root biomass following N fertilization (e.g., Lim et al., 2015) could negatively feedback to long-term foliar C uptake through reductions in tree-scale uptake of water and essential nutrients, such as P. Such responses would further oppose any positive effects on photosynthetic performance by enhanced N availability through reduced stomatal conductance and amplification of foliar nutrient imbalances in the fertilized trees. The observed photosynthetic responses of mature N-poor *P. sylvestris* to increased foliar N contents and their implications on the long-term stand-scale C cycling are discussed below.

### Effects of foliar nutrient contents and needle age on photosynthetic capacity

The greater observed P sensitivity of *J*_max_ than of *V*_cmax_ in the 1-year-old needles of the trees with high N availability (Figure 2) agrees with the results of Niinemets et al. (2001) who collected data from two temperate *P. sylvestris* stands with strong, naturally occurring differences in N availability. Contrary to these findings, a recent meta-analysis by Walker et al. (2014) found the relationship between *J*_max_ and *V*_cmax_ to be unaffected by leaf N, P or *LMA*, suggesting that the capacity parameters may be equally sensitive to P when evaluated for a range of species. Importantly, the varying strength of the capacity limitation by P detected in the current study indicates that assessments of canopy-scale photosynthetic capacity limitation by multiple nutrients must also account for within-canopy variation. Furthermore, while information of needle P contents considerably increased the accuracy of the capacity predictions, the apparent P deficiency may be indicative of general nutrient deficiency, relative to N, in the trees with high N availability.

The photosynthetic capacity of pine needles has been shown to vary seasonally and with needle age (Jach and Ceulemans, 2000; Han et al., 2004, 2008; Op de Beeck et al., 2010; Kolari et al., 2014). We found no needle age-related capacity trends at either plot with the exception of the *J*_max_:*V*_cmax_ variable for which a significant interaction between needle age and canopy position was detected (Tables 2, 3). Notably, neither N nor P alone or their combination was a successful predictor of the photosynthetic capacities of the current-year needles (Figure 1, Figure S2). Troeng and Linder (1982) have shown that the length growth of *P. sylvestris* needles in central Sweden continues until early- to mid-August. Thus, the lack of correlation between photosynthetic capacity and foliar nutrient contents observed in the current study may be due to the current-year needles not having been fully developed at the time of the measurement campaign.

Overall, the higher *V*_cmax_ and *J*_max_ of 1-year-old needles at the fertilized plot support hypothesis #1 with respect to photosynthetic capacity (Tables 2, 3). However, the long-term shoot-scale photosynthetic performance was similar among fertilized and control trees (Figure 4B), thus, contradicting the observed positive biochemical capacity response to N.

### Nutrient-use efficiency and optimality of nitrogen allocation

Our results support hypothesis #2, that greater N availability reduces shoot-scale NUE, with respect to the photosynthetic capacity responses to N but not with respect to the long-term photosynthetic performance. As observed in previous studies (Näsholm and Ericsson, 1990; Nordin et al., 2001), arginine was produced in response to N fertilization but N allocation to arginine can only explain a fraction of the observed between plots difference in the efficiency with which N is used for generating photosynthetic capacity. The improvements in the capacity predictions following inclusion of the P and N^*^P parameters (Figure 2), however, appear to explain the weak N dependencies of photosynthetic capacities at the fertilized plot (Figure 1) and also indicate that properly assessing N allocation requires knowledge of the availability of other resources. These results further suggest that the N allocation pattern at the fertilized plot is non-optimal with the upper canopy needles having more N than they can effectively utilize given their P contents. This supports hypothesis #3 with respect to instantaneous photosynthetic capacity. Interestingly, no vertical variation was observed in the P:N ratio at either plot indicating that P was not preferentially allocated to the upper canopy high-N needles. An upper canopy P limitation could also explain why the capacities did not scale with long-term light availability, i.e., with vertical canopy position, as commonly observed (e.g., Han et al., 2003; Niinemets, 2007; Niinemets et al., 2015). This finding further implies that having lower and mid-canopy needles with reasonably high photosynthetic capacities may have benefits, such as higher long-term canopy-scale LUE, that outweigh the non-optimality of within-canopy N allocation with respect to maximal photosynthetic capacities. Accordingly, it has been previously suggested that due to the photosynthesis of evergreens often being limited by sub-optimal environmental conditions, having foliage capable of high assimilation rates under optimal conditions may offer little long-term benefit (Warren and Adams, 2004; also see Tarvainen et al., 2015). The significant vertical variations in the long-term NUE at both plots suggest that N was non-optimally allocated for maximizing long-term shoot-scale C uptake independently of N availability. Therefore, our results do not support hypothesis #3 with respect to long-term photosynthetic performance. The non-optimality at the N-poor control plot may reflect excess N allocation to lower canopy in response to constraints on leaf morphology (Dewar et al., 2012) or, possibly, resource-use efficiency trade-offs allowing for a higher shoot-scale LUE in the shaded lower canopy at the cost of reduced NUE (Tarvainen et al., 2015).

### Photosynthetic performance modeling

The modeling was rather successful in predicting observed long-term *A*_n_ (Figure 5), especially considering that canopy mean *V*_cmax_ or *J*_max_ were used for all shoots. The overestimation of *A*_n_ in some of the high performance, upper canopy, shoots is likely related to mutual shading of the needles in the continuously measured shoots leading to a lower per unit needle area light interception than indicated by the sensor reading. The effect would be weaker at low *Q* when the incoming light is mostly diffuse and at very high, but rare, *Q* when there is an excess of light. Because the needles were separated to avoid shading when the capacity parameters were determined, the model calculations do not account for this effect. Additionally, it should be noted that the modeling was based on *C*_i_ rather than chloroplast CO_2_ concentrations, which has been shown to affect the predictive power of the Farquhar et al. (1980) photosynthesis model (Niinemets et al., 2009). The relatively higher CO_2_ assimilation rates in the upper canopy shoots, due to higher light availability, could lead to the difference between *C*_i_ and chloroplast CO_2_ concentrations becoming considerable in these locations. In such cases, using *C*_i_-based modeling would likely contribute toward overestimation of *A*_n_. Furthermore, both nutrient availability (Warren, 2004; Bown et al., 2009) and tree height (Han, 2011) can affect mesophyll conductance suggesting that some of the apparent between-plots capacity differences observed in this study could also result from differences in mesophyll conductance. The modeling also did not account for the reduction in respiration rate in light (e.g., Way and Yamori, 2014), which would push the model toward underestimating *A*_n_ during daytime hours. However, given the relative magnitudes of respiration and assimilation rates, an adjustment of the respiration term would have only a negligible impact on the predicted long-term *A*_n_. Despite of these potential confounding factors, we found the accuracy of the model to be acceptable for estimating long-term *A*_n_ losses due to P deficiency and N allocation to arginine.

### Phosphorus and arginine effects on needle- and canopy-scale carbon uptake

Assessing the capacity limitation of 1-year-old fertilized upper canopy needles, with mean *N*_a_ of 6.5 g N m^−2^ s^−1^, by extrapolating the capacity—*N*_a_ relationships from the control plot suggested that *V*_cmax_ was reduced by 8.5% and *J*_max_ by 27%. Similar results were presented by Loustau et al. (1999) who found that P deficiency constrained *A*_n_ through *V*_cmax_ at high light availability and through *J*_max_ at low light availability in 2-year-old pot-grown *Pinus pinaster* seedlings. Despite the considerable apparent limitation of *V*_cmax_ and *J*_max_ in the current study, the predicted long-term performance loss in the 1-year-old fertilized upper canopy shoots was estimated to *c*. 10% per unit needle area. Furthermore, because capacity limitation was only observed in 1-year-old needles and would occur predominantly in the upper canopy, the reduction of canopy-scale C uptake at the fertilized plot by P and arginine appears to be modest and, thus, our findings do not support hypothesis #4. Similarly, Op de Beeck et al. (2010) showed that seasonal and age-related photosynthetic capacity variation had negligible effects on model predictions of annual canopy-scale gas exchange of a temperate *P. sylvestris* stand experiencing high N deposition rates.

### Constraints of long-term canopy-scale carbon assimilation

The similarity of needle area-based LUE between the plots (Figure 3B) after 8 years of heavy N-fertilization, suggests that any long-term differences in canopy-scale C assimilation will depend on differences in total canopy-scale leaf area and light interception rather than foliar nutrient contents as has also been suggested previously (Brix, 1971; Linder and Axelsson, 1982). As observed in previous studies of N fertilization effects on N-limited forests (see the review by Hyvönen et al., 2007), leaf area of the fertilized plot has increased in response to N addition and was *c*. 13 % greater than at the control plot in 2013 (Lim et al., 2015). Nitrogen availability has been observed to affect shoot structure and, thereby, the light interception efficiency in *P. sylvestris* (Niinemets et al., 2002). However, the lack of between plots variation in the vertical LUE patterns (Figure 3B) indicates that needle-area based C assimilation was insensitive to any such potential changes in shoot structure. This agrees with the suggestion by Lim et al. (2015) that the impact of fertilization on canopy-scale light absorption at the current site was rather small and mainly due to leaf area responses to increasing N availability. Moreover, the positive effect of the observed leaf area increase on the canopy-scale C uptake was likely reduced by lower average *Q* per unit needle area caused by increased within-canopy shading. The smallish between plots difference in expected canopy-scale light interception is relevant for stand-scale C cycling considering that Lim et al. (2015) observed a 27 ± 7% higher aboveground primary production (ANPP) at the fertilized plot compared to the control plot. Increasing aboveground productivity is a commonly observed response to N addition in boreal conifers (e.g., Axelsson and Axelsson, 1986; Linder, 1987), and in agreement with previous studies (e.g., Linder and Axelsson, 1982; Axelsson and Axelsson, 1986; Gower et al., 1994), Lim et al. (2015) suggested that the ANPP difference resulted mainly from changes in C partitioning to aboveground and belowground biomass pools. The current study provides additional evidence of the importance of partitioning shifts by showing that photosynthetic performance per unit needle area was not enhanced in response to the N fertilization and, thus, increased canopy-scale C uptake could only account for a portion of the observed ANPP difference.

In summary, our results suggest that in mature stands mutual shading largely negates the canopy-scale C uptake benefit of having foliage with high photosynthetic capacities, i.e., high *N*_a_. The light limitation also reduces the benefit of using captured C for construction of additional leaf area; rather the most beneficial strategy appears to be to have low- and mid-canopy foliage capable of reasonable C uptake and to grow in height to maximize light interception. Many of the large-scale fertilization trials that have concluded that boreal forests are N-limited have, however, been carried out in young stands. Such stands are likely to be able to greatly increase their light capture and C uptake by increasing the rate of leaf area expansion, and by having foliage with greater photosynthetic capacity, prior to canopy closure. In such cases, limitations to N availability could considerably reduce the canopy-scale C uptake and, thus, growth.

## Conclusions

Our results suggest that the canopy-scale photosynthetic performance of the studied N-poor stand was not greatly limited by N, or by capacity constraints in response to P deficiency or arginine synthesis following N addition. Rather, the long-term photosynthetic limitation was caused by the existing foliar nutrients not being utilized to their full potential due to the light availability constraints affecting most of the canopy. The weak N dependency of long-term foliar C uptake suggests that models predicting stand-scale responses to changes in N availability should include robust representations of N effects on within-tree C partitioning and light-use efficiency as well as availability patterns, rather than being reliant on N-based estimates of photosynthetic capacity. In this context, it would be particularly interesting to explore the potential shift in fertilization response from maintaining high NUE, as the additional N is spread among more needles, in expanding stands to prioritizing high needle-scale LUE in closed stands where the additional N must be allocated within the existing canopy structure. Finally, our results suggest that there is a strong need for studies investigating photosynthetic acclimation responses under sub-optimal environmental conditions, such as low light availabilities and nutrient imbalances, likely experienced by the majority of the foliage within a canopy.

## Author contributions

LT, ML, and GW designed the experiment. LT, ML, MR, and GW performed the field measurements. All authors contributed to the analysis and interpretation of the results. LT wrote the manuscript with ML, MR, TN, and GW providing editorial advice. All authors read and approved the final manuscript.

## Funding

This study was supported by The Kempe foundations, The Swedish University of Agricultural Sciences (TC4F and Bio4E), The Strategic Research Area “Biodiversity and Ecosystem Services in a Changing Climate” (BECC) and the research councils: The Swedish Research Council for Environment, Agricultural Sciences and Spatial Planning, The Swedish Research Council and The Swedish Governmental Agency for Innovation Systems.

### Conflict of interest statement

The authors declare that the research was conducted in the absence of any commercial or financial relationships that could be construed as a potential conflict of interest.

## Supplementary material

Image S1 **Photograph of an automated cuvette used for continuous measurements of shoot-scale gas exchange**.

Figure S1 **Vertical variation in fraction visible sky (openness) in the studied ***Pinus sylvestris*** plots**.

Figure S2 **The impact of including both nitrogen and phosphorus in the predictions of maximum carboxylation rate (***V***_**cmax**_) and maximum electron transport rate (***J***_**max**_) at 25°C in current-year ***Pinus sylvestris*** needles**.

Table S1 **Variation in needle physical and chemical properties with canopy position (upper, mid, lower) in 1-year-old ***Pinus sylvestris*** shoots used in continuous shoot-scale net gas exchange measurements**.

Table S2 **Repeated measures ANOVA statistics for between plots and age-related variation in the needle properties in the upper canopy shoots including all age three classes presented in Tables 1, 2**.
