# Supplementary material for: Increased Needle Nitrogen Contents Did Not Improve Shoot Photosynthetic Performance of Mature Nitrogen-Poor Scots Pine Trees
Source: Front Plant Sci. 2016 Jul 20;7:1051. doi: 10.3389/fpls.2016.01051 (PMC4951524; doi:10.3389/fpls.2016.01051)
Supplement: Image S1 — Photograph of an automated cuvette used for continuous measurements of shoot-scale gas exchange. [file DataSheet1.DOCX]

***Supplementary Material***

**Increased needle nitrogen contents did not improve shoot photosynthetic performance of mature nitrogen-poor Scots pine trees**

**Lasse Tarvainen^1,*^, Martina Lutz^2^, Mats Räntfors^2^, Torgny Näsholm^1^, Göran Wallin^2^**

*** Correspondence:** Lasse Tarvainen, lasse.tarvainen@slu.se

**Image S1** A transparent temperature-controlled cuvette used for automated measurements of the long-term gas exchange of Scots pine shoots. The light sensor (the black cylinder with a white cap to the left of the cuvette) is located outside the cuvette but is < 5 cm away. Heated tubing connects the reference and sample (i.e. from inside of the ventilated cuvette) air streams to infrared gas analyzers located in a nearby hut, *c*. 20 m away. Photo: Lasse Tarvainen.


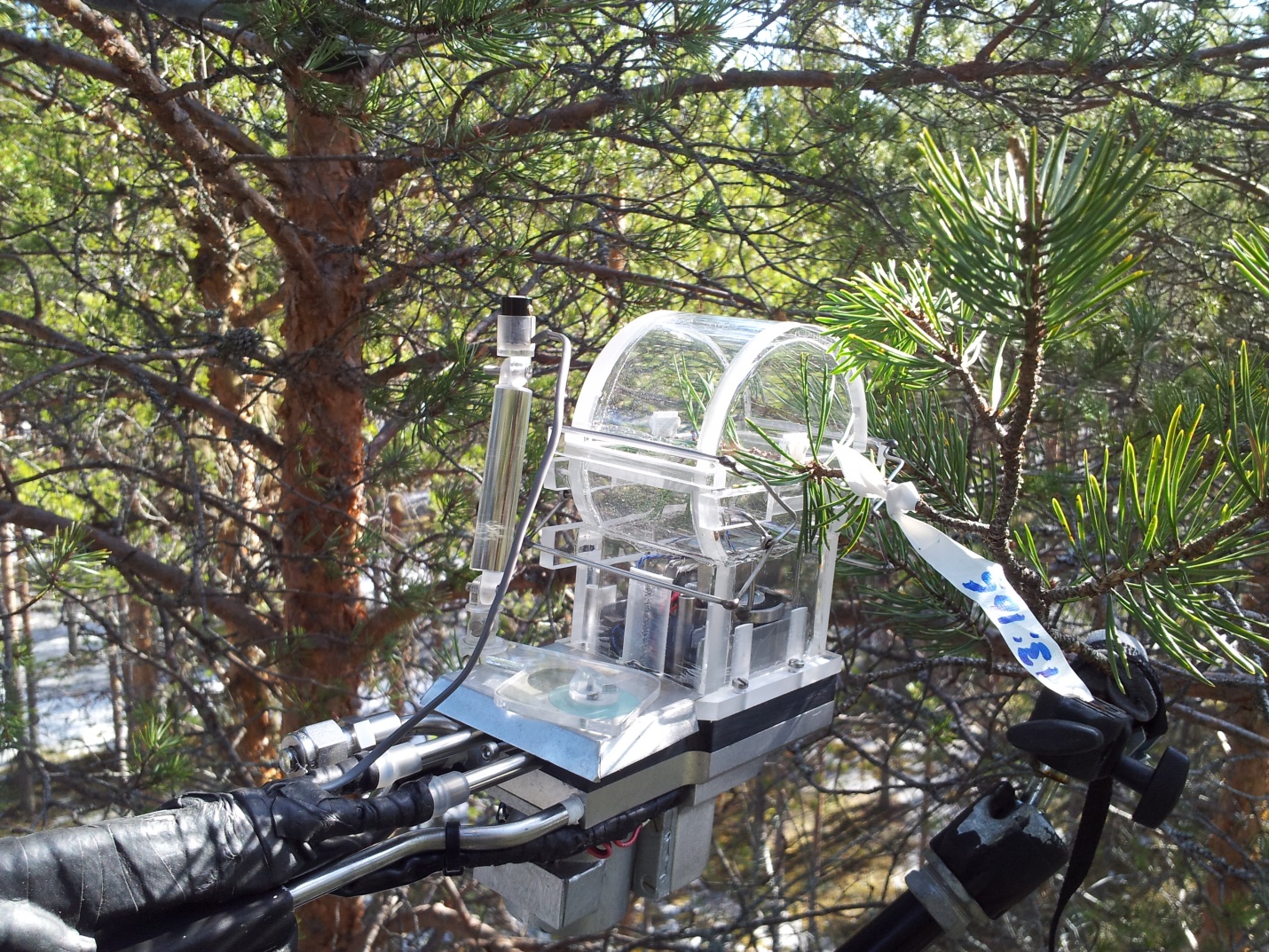


**Table S1** Variation in needle physical and chemical properties with canopy position (upper, mid, lower) in one-year-old *Pinus sylvestris* shoots used in continuous shoot-scale net gas exchange measurements at the fertilized (F) and control (C) plots in August 2013 (mean ± SD, *n* = 2-3).

|  |  |  | Whole needles | | Mid-sections (estimated) | |
| --- | --- | --- | --- | --- | --- | --- |
|  |  |  | Plot | | | |
|  | Unit | Position | F | C | F | C |
| Height | m | upper | 15.5 ± 0.0 | 14.8 ± 0.6 |  |  |
|  |  | mid | 13.1 ± 0.7 | 13.1 ± 0.1 |  |  |
|  |  | lower | 10.9 ± 0.7 | 11.4 ± 0.1 |  |  |
|  |  |  |  |  |  |  |
| *Q* | µmol m^-2^ s^-1^ | upper | 300 ± 65 | 233 ± 52 |  |  |
|  |  | mid | 152 ± 6 | 151 ± 77 |  |  |
|  |  | lower | 52 ± 25 | 85 ± 24 |  |  |
|  |  |  |  |  |  |  |
| *LMA* | g m^-2^ | upper | 360 ± 8 | 283 ± 27 | 316 ± 7 | 249 ± 23 |
|  |  | mid | 296 ± 9 | 270 ± 12 | 259 ± 8 | 236 ± 11 |
|  |  | lower | 257 ± 26 | 223 ± 15 | 225 ± 23 | 196 ± 13 |
|  |  |  |  |  |  |  |
| *N*_a_ | g N m^-2^ | upper | 7.6 ± 0.0 | 3.1 ± 0.0 | 6.9 ± 0.0 | 2.7 ± 0.0 |
|  |  | mid | 6.5 ± 0.5 | 2.9 ± 0.4 | 5.7 ± 0.4 | 2.5 ± 0.4 |
|  |  | lower | 5.4 ± 0.1 | 2.5 ± 0.3 | 4.8 ± 0.1 | 2.2 ± 0.3 |
|  |  |  |  |  |  |  |
| *N*_m_ | mg g^-1^ | upper | 21.2 ± 0.4 | 11.0 ± 1.0 |  |  |
|  |  | mid | 21.9 ± 0.8 | 10.7 ± 1.2 |  |  |
|  |  | lower | 21.3 ± 1.8 | 11.2 ± 0.8 |  |  |
|  |  |  |  |  |  |  |
| *P*_a_ | g P m^-2^ | upper | 0.67 ± 0.03 | 0.46 ± 0.03 | 0.58 ± 0.03 | 0.40 ± 0.02 |
|  |  | mid | 0.59 ± 0.04 | 0.43 ± 0.02 | 0.52 ± 0.04 | 0.37 ± 0.02 |
|  |  | lower | 0.53 ± 0.02 | 0.36 ± 0.07 | 0.47 ± 0.02 | 0.31 ± 0.06 |
|  |  |  |  |  |  |  |
| *P*_m_ | mg g^-1^ | upper | 1.9 ± 0.1 | 1.6 ± 0.01 |  |  |
|  |  | mid | 2.0 ± 0.1 | 1.6 ± 0.2 |  |  |
|  |  | lower | 2.1 ± 0.1 | 1.6 ± 0.2 |  |  |
|  |  |  |  |  |  |  |
| P:N | g P g^-1^ N | upper | 0.09 ± 0.00 | 0.15 ± 0.01 |  |  |
|  |  | mid | 0.09 ± 0.00 | 0.15 ± 0.03 |  |  |
|  |  | lower | 0.10 ± 0.00 | 0.014 ± 0.00 |  |  |

*Q* = shoot-incident photosynthetic photon flux density; LMA = leaf mass per unit area; *N*_a_ and *N*_m_ = needle nitrogen content per unit area and mass, respectively; *P*_a_ and *P*_m_ = needle phosphorus content per unit area and mass, respectively. Mid-sections; variable estimates if analysed analogously to the needles used for capacity measurements (Table 1, see material and methods for details).

**Table S2** Repeated measures ANOVA statistics for between plots and age-related variation in the needle properties in the upper canopy shoots including all age classes presented in tables 1 and 2.

| Variable | Age | Plot | Age*Plot |
| --- | --- | --- | --- |
| Height | n.s. | n.s. | n.s. |
|  |  |  |  |
| Openness | n.s. | n.s. | n.s. |
|  |  |  |  |
| *LMA* | n.s. (C)  <0.001 (F) | n.s. (0)  n.s. (1)  0.021 (2) | 0.037 |
|  |  |  |  |
| *N*_a_ | n.s. (C)  <0.001 (F) | <0.001 | 0.008 |
|  |  |  |  |
| *N*_m_ | n.s. | <0.001 | n.s. |
|  |  |  |  |
| *P*_a_ | n.s. | n.s. | n.s. |
|  |  |  |  |
| *P*_m_ | n.s. | n.s. | n.s. |
|  |  |  |  |
| *P*:N | n.s. | 0.008 | n.s. |
|  |  |  |  |
| α | n.s. | n.s. | n.s. |
|  |  |  |  |
| *V*_cmax_ | 0.011 | n.s. | n.s. |
|  |  |  |  |
| *J*_max_ | n.s. | n.s. | n.s. |
|  |  |  |  |
| *J*_max_:*V*_cmax_ | n.s. | n.s. | n.s. |
|  |  |  |  |
| *V*_cmax_:N | n.s. | 0.002 | n.s. |
|  |  |  |  |
| *J*_max_:N | n.s. | 0.001 | n.s. |
|  |  |  |  |
| *V*_cmax_:P | n.s. | n.s. | n.s. |
|  |  |  |  |
| *J*_max_:P | n.s. | n.s. | n.s. |

C = control plot; F = fertilized plot; 0, 1 and 2 = needle age in years. Simple main effects were tested for where significant interactions were detected. Bonferroni corrections were applied for analyses of multiple correlations, n.s. = not significant (*P* > 0.05).

**Figure S1** Vertical variation in diffuse non-interceptance (openness) measured at shoot locations at the fertilized (F, filled symbols, solid line) and control (C, open symbols, dashed line) plots in the studied *Pinus sylvestris* stand. Squares = current-year needles, circles = one-year-old needles.

**Figure S2** Predictions of maximum carboxylation rate (*V*_cmax_) and maximum electron transport rate (*J*_max_) at 25 °C in *Pinus sylvestris* based on i) needle nitrogen content per unit area, *N*_a_, (N: open circles) and ii) *N*_a_ and needle phosphorus content per unit area, *P*_a_, (N,P: filled circles) of current-year needles compared with measured rates. *V*_cmax_ on the **(A)** fertilized and **(B)** control plots; *J*_max_ on the **(C)** fertilized and **(D)** control plots.
